# Le fibromatosis colli ou torticolis congénital: son diagnostic et sa prise en charge à propos de deux cas

**DOI:** 10.11604/pamj.2015.22.74.7836

**Published:** 2015-09-30

**Authors:** Mazamaesso Tchaou, Plaodezina Essobozou Pegbessou, Lantam Sonhaye, Patricia Yékpé Ahouanssou, Abdoulatif Amadou, Beresa Kolou, Lidi Bessi Kama, Nouhou Mahamadou Garba, Lama Kegdigoma Agoda Koussema, Koffi N'dakéna

**Affiliations:** 1 Service de Radiologie et Imagerie médicale CHU Sylvanus Olympio, Lomé, Togo; 2 Service d'ORL CHU Sylvanus Olympio, Lomé, Togo; 3 Service de Radiologie et Imagerie médicale CHU Campus, Lomé, Togo; 4 Service de Radiologie et Imagerie médicale CHNHU HKM, Cotonou, Bénin; 5 Service de Pédiatrie CHU Sylvanus Olympio, Lomé, Togo

**Keywords:** Fibromatosis colli, torticolis congénital, sterno-cléido-mastoïdien, échographie, Fibromatosis colli, congenital torticollis, sternocleidomastoid muscle, echography

## Abstract

Le fibromatosis colli (FC) est pseudotumeur rare du muscle sterno-cléido-mastoïdien (SCM), à l'origine d'un torticolis dit congénital chez le nouveau-né ou le nourrisson. Le mécanisme étio-pathogénique de sa survenue est sujet à controverse. Son diagnostic fait appel à l’échographie qui permet de mettre en évidence un épaississement caractéristique du muscle. Nous rapportons deux cas diagnostiqués par l’échographie avec pour un cas une notion de malposition intra-utérine et pour l'autre cas une absence totale de malposition et de traumatisme obstétrical qui pourtant est évoqué comme élément du mécanisme de survenue du FC.

## Introduction

Décrite pour la première fois par l'allemand Hulbert comme torticolis tumorale du muscle sterno-cléido-mastoïdien (SCM), le fibromatosis colli (FC) est une lésion bénigne du muscle SCM, liée à une fibrose congénitale, se manifestant cliniquement par une tuméfaction latéro-cervicale et un torticolis [1, 2]. Son étiologie et le mécanisme physio-pathologique conduisant à la fibrose du muscle ne sont pas claire et sont toujours sujet à controverse. Malgré un diagnostic facilement évoqué à la clinique, l'imagerie en coupe, avec comme examen de premier choix l’échographie reste utile pour en faire le diagnostic de certitude en éliminant d'autres causes de torticolis congénital et de masse latéro-cervicale, mais aussi d'en assurer la surveillance lors de l’évolution. Nous rapportons deux cas de FC spontanément et rapidement régressifs en 3 et 4 mois chez deux nourrissons diagnostiqués et surveillés par l’échographie.

## Patient et observation

### Cas 1

**Figure 1:**
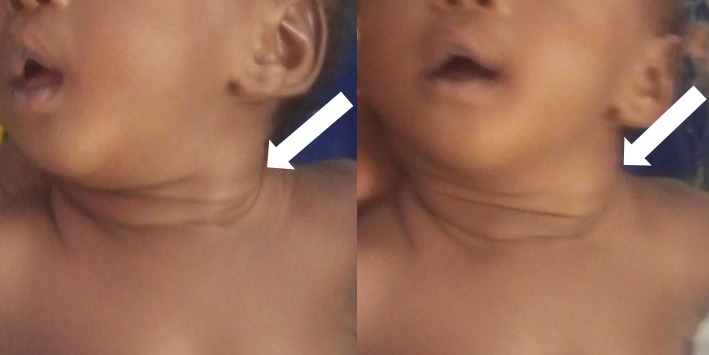
Nourrisson de sexe féminin de 6 semaines, née par césarienne pour présentation de siège, présentant une tuméfaction latéro-cervicale gauche (flèche blanche) et une déviation de la tête vers le côté droit

**Figure 2:**
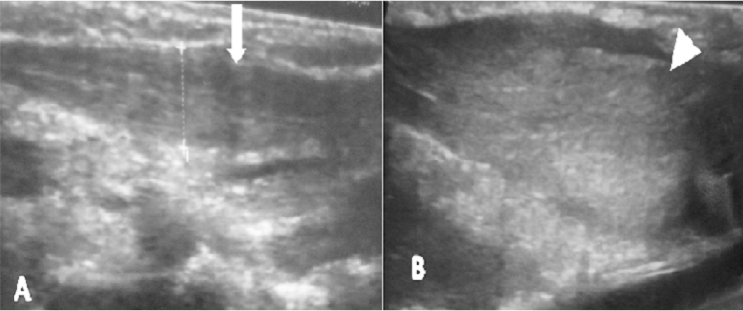
Coupes échographiques longitudinales du cou du nourrisson de 6 semaines, à l'aide d'une sonde linéaire de 12MHZ, montrant les muscles sterno-cléido-mastoïdiens (SCM) droit (A) et gauche (B), avec un épaississement fusiforme hyper-échogène du corps du muscle SCM droit (tête de flèche blanche) comparativement au muscle du côté opposé (flèche blanche), avec conservation de son aspect fibrillaire

Nourrisson de sexe féminin de 6 semaines, adressée par le service de chirurgie pédiatrique au service de radiologie et imagerie médicale du Centre Hospitalier Universitaire SylvanusOlympio (CHUSO) de Lomé (Togo) pour exploration échographique d'une tuméfaction latéro-cervicale gauche (Figure 1). Née par césarienne à 38 semaines d'aménorrhées (SA) pour présentation de siège chez une primipare à l'issue d'une grossesse sans incident, son poids de naissance était de 3,100 Kg pour une taille de 52 cm. Il n'existait aucun antécédent familial pathologique et pas de lien de consanguinité entre les parents. Dans l'histoire, la tuméfaction avait été constatée déjà à 4 semaines de vie, l'attention de la maman ayant été attirée par une déviation permanente de la tête du bébé du côté droit. Il n'y avait pas de notion de fièvre, ni de traumatisme. L’échographie réalisée à l'aide d'une sonde linéaire de 12 MHZ notait un épaississement fusiforme du corps du muscle SCM gauche d'aspect hyper-échogène par rapport au reste du muscle et comparativement au côté opposé, avec conservation toute fois de l'aspect fibrillaire du muscle (Figure 2). Le reste de l'examen échographique du cou était normal. Devant cet aspect échographique et clinique, le diagnostic de FC avait été retenu. Aucun traitement médicamenteux ou chirurgical n'avait été prescrit. Il avait été conseillé à la maman de porter le bébé le plus souvent possible au dos, la face tournée vers le côté de la lésion. Lors des suivis réalisés chaque mois, nous avons observé une régression progressive de la tuméfaction et du torticolis, jusqu’à leur disparition complète au bout du quatrième mois.

### Cas 2

**Figure 3:**
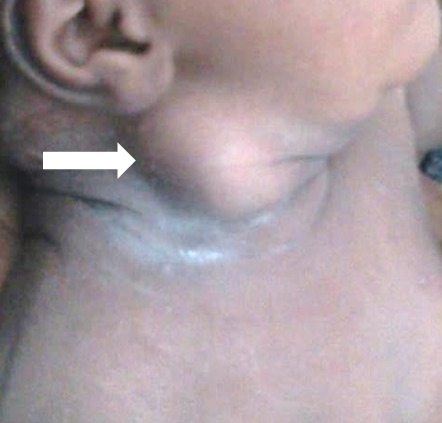
Nouveau-né de sexe masculin de 3 semaines, présentant une tuméfaction latéro-cervicale droite (flèche blanche)

**Figure 4:**
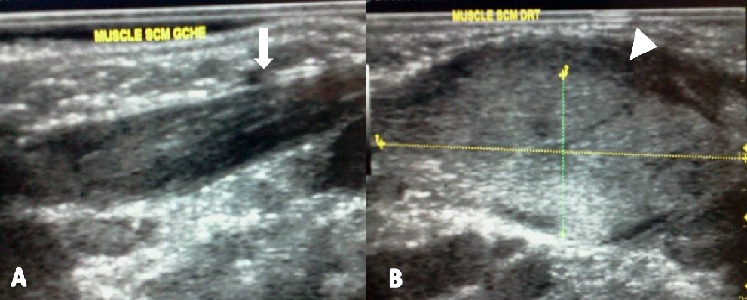
Coupes échographiques longitudinales du cou du nouveau-né de 3 semaines, à l'aide d'une sonde linéaire de 12MHZ, montrant les muscles sterno-cléido-mastoïdiens (SCM) gauche (A) et droit (B), avec un épaississement fusiforme hyper-échogène du corps du muscle SCM droit (tête de flèche blanche) comparativement au muscle du côté opposé (flèche blanche), avec conservation de son aspect fibrillaire

Nouveau-né de sexe masculin de 3 semaines, adressé par le service d'Oto-Rhino-Laryngologie (ORL) au notre pour exploration échographique d'une tuméfaction latéro-cervicale droite (Figure 3). Né d'un accouchement normal à 38 SA, il avait un poids de naissance de 3,000 Kg pour une taille de 50 cm. Il n'y avait pas d'anomalie de présentation et l'accouchement était eutocique. Il n'existait ni antécédent familiale pathologique ni lien de consanguinité entre les parents. La tuméfaction s'accompagnait d'un torticolis, avec une déviation de la tête vers le côté gauche. Il n'y avait pas de notion de fièvre, ni de traumatisme. L’échographie réalisée à l'aide d'une sonde linéaire de 12 MHZ notait un épaississement fusiforme hyper-échogène du corps du muscle SCM droit comparativement au muscle du côté opposé, avec conservation de l'aspect fibrillaire du muscle (Figure 4). Le reste de l'examen échographique du cou était normal. Devant cet aspect échographique et clinique, le diagnostic de FC avait été retenu et le bébé avait bénéficié des mêmes mesures physiques que dans le premier cas. La régression totale de la tuméfaction et du torticolis avait été observée au quatrième mois.

## Discussion

La prévalence du FC est estimée à 0,3 - 2% des naissances, avec une prédominance masculine. Le côté droit semble plus fréquemment touché, dans les proportions de 60 - 75%. L'atteinte bilatérale est rare, environ 2 - 8% des cas [2]. Sa prévalence en Afrique est inconnue [3] et très peu de publications scientifiques existent sur des cas africains, en dehors de la série de Abdur-Rahman LO et al de 15 cas colligés en 10 ans (1999-2008) à l'hôpital universitaire d'Ilorin au Nigéria [4]. Selon les mêmes auteurs, il existe un retard à la consultation en Afrique, comme c'est le cas dans beaucoup d'autres maladies. En effet, selon eux, nombreux parents présentent leurs enfants après 3 mois à cause du fait qu'ils présument que la position anormale de la tête de l'enfant avant cet âge serait due à la non acquisition de la tenue de la tête qui normalement ne s'acquière qu’à 3 mois dans le développement psychomoteur normal de l'enfant. Certains cas selon toujours les mêmes auteurs sont constatés en premier par les grand-mères lors des massages traditionnels du corps du bébé. Pour nos deux cas, la consultation a été précoce dès la constatation de l'anomalie respectivement à 6 et à 3 semaines de vie, ceci témoigne du changement de comportement dans la population africaine surtout en milieu urbain. Tumeur bénigne selon l'Organisation Mondiale de la Santé (OMS), le FC est classé dans la catégorie des proliférations fibroblastiques bénignes selon la classification OMS 2002 des tumeurs des tissus mous [5]. Son étiopathogénie n'est pas connue à ce jour. Elle reste encore un mystère. En effet, ses causes sont encore débattues, les théories variant d'un auteur à l'autre. La plus courante étant celle qui lie l'anomalie du SCM à une fibrose et contracture du muscle secondaire à un syndrome des loges et à des lésions ischémiques favorisées par une malposition f'tale intra utérine [6]. Une autre théorie serait plutôt en faveur des traumatismes ou microtraumatismes du SCM lors d'un accouchement laborieux [7]. Ces deux mécanismes peuvent être intriqués, la malposition entrainant un accouchement laborieux. Dans notre premier cas il existe effectivement une malposition intra-utérine, mais pas de traumatisme obstétrical. Pour le second cas il n'existe ni malposition intra utérine ni notion d'accouchement difficile, ce qui confirme le mystère autour de l’étiopathogénie du FC, même si les lésions anatomopathologiques retrouvées sur les pièces de biopsie, à savoir un remplacement des fibres musculaires par des amas de tissus fibrosés fait de cellules fibroblastiques matures confirme la fibrose [8]. L’échographie nous a permis comme pour plusieurs auteurs, de poser le diagnostic de FC devant l'aspect typique d'un épaississement fusiforme du muscle SCM [7, 8]. C'est l'examen de choix, reconnu par tous les auteurs du fait de son accessibilité aisée, son coût faible, et son caractère non irradiant [7]. Les autres techniques d'imagerie en coupe notamment la tomodensitométrie (TDM) et l'imagerie par résonnance magnétique (IRM) peuvent également mettre en évidence l’épaississement du muscle, mais il s'agit de moyens peu accessibles, coûteux et irradiant pour la TDM [7]. Certains auteurs estiment que l'examen clinique suffit au diagnostic et qu'aucune investigation supplémentaire ne devrait être réalisée en routine [4], mais ils reconnaissent néanmoins l'utilité de l’échographie pour éliminer d'autres étiologies lorsque l'aspect clinique est atypique. La prise en charge du FC fait appel essentiellement chez les nouveaux nés et les nourrissons à la physiothérapie. Dans les deux cas de notre étude, les mamans ont été conseillées et encouragées à porter leurs bébés au dos avec la face tournée vers le côté de la lésion, comme elles le font traditionnellement dans notre milieu. Cette méthode a été décrite déjà par l’équipe de l'Hôpital universitaire d'Ilorin au Nigeria [4]. Le traitement chirurgical est rarement utile, moins de 5% des cas lorsque le diagnostic est précoce mais la proportion peut atteindre la moitié des cas lorsque le diagnostic est fait au-delà du sixième mois [9]. L’évolution du FC se fait même en l'absence de traitement, vers la régression spontanément en 4 à 6 mois [10], quelques fois même beaucoup plus tôt à 3 mois d’âge [7]. Cette évolution spontanée peut être facilitée et/ou accélérée par la physiothérapie qui pour nos cas à consister à encourager les mamans à continuer une technique de port de bébé au dos traditionnellement utilisée dans notre milieu.

## Conclusion

Le fibromatosis colli est relativement rare, sa fréquence en Afrique reste à établir. Son diagnostic associe la clinique et l’échographie qui permet d’éliminer d'autre cause de torticolis chez le nouveau-né et le nourrisson. La physiothérapie active peut être valablement remplacée par le port traditionnel du bébé au dos, tête tourné du côté ipsilatéral à la lésion qui permet d'obtenir une régression rapide et spontanée de la lésion en 3-4 mois.
